# Synergistic activation of human pregnane X receptor by binary cocktails of pharmaceutical and environmental compounds

**DOI:** 10.1038/ncomms9089

**Published:** 2015-09-03

**Authors:** Vanessa Delfosse, Béatrice Dendele, Tiphaine Huet, Marina Grimaldi, Abdelhay Boulahtouf, Sabine Gerbal-Chaloin, Bertrand Beucher, Dominique Roecklin, Christina Muller, Roger Rahmani, Vincent Cavaillès, Martine Daujat-Chavanieu, Valérie Vivat, Jean-Marc Pascussi, Patrick Balaguer, William Bourguet

**Affiliations:** 1 Inserm U1054, Montpellier 34090, France; 2 CNRS UMR5048, Centre de Biochimie Structurale, Montpellier 34090, France; 3 Université de Montpellier, Montpellier 34090, France; 4 IRCM, Institut de Recherche en Cancérologie de Montpellier, Montpellier 34298, France; 5 Inserm, U1194, Montpellier 34298, France; 6 ICM, Institut régional du Cancer de Montpellier, Montpellier 34298, France; 7 Inserm U1040, Montpellier 34295, France; 8 Inserm U661, Montpellier 34094, France; 9 CNRS UMR5203, Institut de Génomique Fonctionnelle, Montpellier 34094, France; 10 NovAliX, Illkirch 67400, France; 11 INRA UMR 1331, TOXALIM, Sophia-Antipolis 06903, France; 12 CHU de Montpellier, Institut de Recherche en Biothérapie, Montpellier 34295, France

## Abstract

Humans are chronically exposed to multiple exogenous substances, including environmental pollutants, drugs and dietary components. Many of these compounds are suspected to impact human health, and their combination in complex mixtures could exacerbate their harmful effects. Here we demonstrate that a pharmaceutical oestrogen and a persistent organochlorine pesticide, both exhibiting low efficacy when studied separately, cooperatively bind to the pregnane X receptor, leading to synergistic activation. Biophysical analysis shows that each ligand enhances the binding affinity of the other, so the binary mixture induces a substantial biological response at doses at which each chemical individually is inactive. High-resolution crystal structures reveal the structural basis for the observed cooperativity. Our results suggest that the formation of ‘supramolecular ligands' within the ligand-binding pocket of nuclear receptors contributes to the synergistic toxic effect of chemical mixtures, which may have broad implications for the fields of endocrine disruption, toxicology and chemical risk assessment.

## Abstract (web summary)

Endocrine-disrupting chemicals act on nuclear hormone receptors, such as PXR. Here, Delfosse *et al*. show how two such chemicals interact with each other in the PXR ligand-binding pocket, forming a so-called supramolecular ligand that is a more potent PXR activator than each of the two chemicals alone.

## Graphical abstract

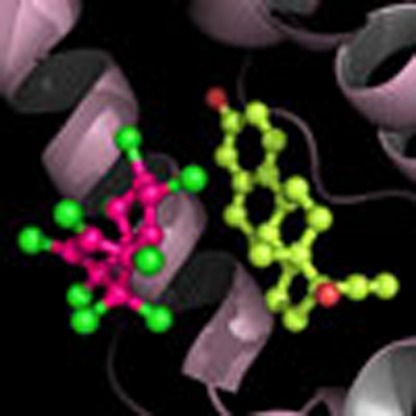

External compounds (xenobiotics) to which humans are continuously exposed include environmental pollutants, drugs or dietary components. Many of them belong to the structurally heterogeneous group of endocrine-disrupting chemicals (EDCs) that trigger adverse health effects by mimicking or antagonizing the action of endogenous signalling molecules^1^,^2^,^3^. More than 20 years of experimental and epidemiological studies have highlighted the pivotal role of nuclear receptors (NRs) in transducing many of the harmful effects of EDCs^4^,^5^. NRs belong to a large family of evolutionarily related transcription factors that control complex gene networks, resulting in profound physiological changes^6^. They contain a ligand-binding domain (LBD) that responds to a wide variety of endogenous hormonal and metabolic ligands. The endocrine-disrupting action of chemicals relies mostly on their ability to substitute for natural ligands and deregulate NR signalling, causing reproductive, proliferative and metabolic disorders^7^,^8^,^9^. In addition, human exposure to mixtures of xenobiotics can induce unpredictable additive, antagonistic or synergistic adverse effects^10^. Yet, the molecular mechanisms underlying these cocktail effects are largely unknown.

To explore the outcome of combined exposure to chemicals and establish a detailed mechanistic understanding of this emerging paradigm for EDC action, we focused our attention on the xenoreceptor PXR (pregnane X receptor; NR1I2) which has been identified by the US Environmental Protection Agency ToxCast's program as a major front-line target of chemicals. This NR is a key regulator of the body's defense against foreign substances. It forms heterodimers with the retinoid X receptor (RXR) and binds to PXR responsive elements (PXRE) in the regulatory regions of target genes. Upon activation by xenobiotics (for example, bisphenol-A, organophosphate pesticides, alkylphenols, rifampicin), PXR interacts with coactivators, such as the steroid receptor coactivator-1 (SRC-1), and transcriptionally upregulates major detoxification genes such as the phase I cytochrome P450 enzyme CYP3A4 (ref. 11), which metabolizes more than half of all drugs in clinical use. On the other hand, the interaction of PXR with EDCs has been linked to an increased risk of cardiovascular^12^ and metabolic^13^ diseases.

Here, using compound screening followed by extensive functional analysis, we demonstrate that the combined use of the pesticide *trans*-nonachlor (TNC) and the active component of contraceptive pills, 17α-ethinylestradiol (EE2), produces synergistic effects on PXR activation and expression of its endogenous target gene *CYP3A4*. Biophysical characterization reveals that EE2 and TNC bind cooperatively to PXR and that the binary mixture has considerably improved functional properties over each of the compounds alone. Crystallographic analysis shows that reciprocal stabilization of the compounds in the ligand-binding pocket (LBP) of the receptor accounts for the enhanced efficacy and potency of the chemical mixture. We therefore propose the concept of a ‘supramolecular ligand' that defines a molecular assembly consisting of two or more compounds that interact with each other inside the LBP of a receptor, resulting in the creation of a new entity with improved functional characteristics in regard to those of its individual components.

## Results

### Synergistic activation of PXR by EE2 and TNC

**Figure 1:**
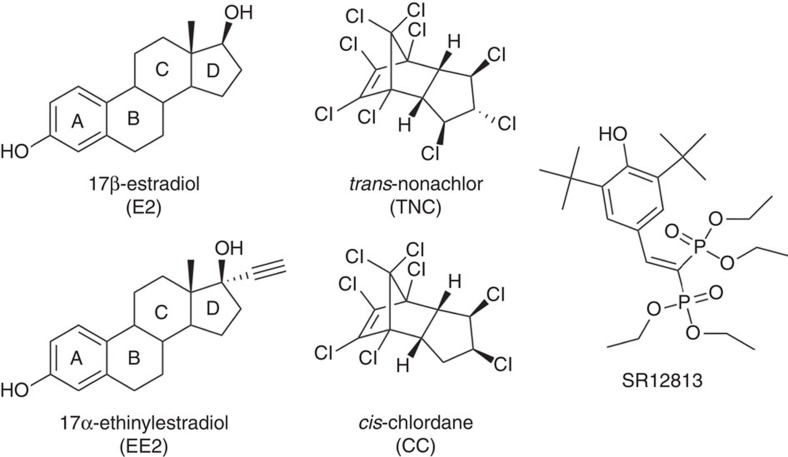
Chemical structures of compounds used in this study.

**Figure 2:**
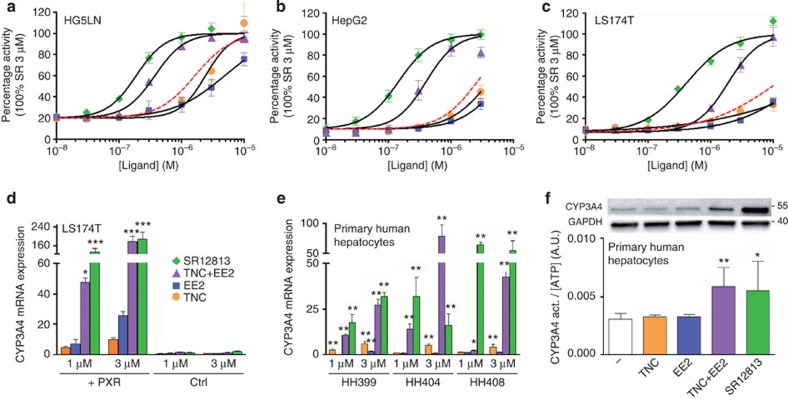
EE2 and TNC activate PXR in a synergistic fashion. HG5LN GAL4-PXR-LBD **(a)**, HepG2-PXR 3A4-Luciferase **(b)**, and LS174T-PXR 3A4-Luciferase **(c)** cells were exposed to different concentrations of SR12813, TNC and EE2 either alone or in combination. Assays were performed in quadruplicate in at least three independent experiments and data are expressed as mean (±s.e.m.). Red dashed lines represent the theoretical activation curves obtained for the additive combination of EE2 and TNC activities calculated using the Bliss independence model^19^. (**d**) RT-qPCR analysis of CYP3A4 mRNA expression in control (Ctrl) or PXR overexpressing LS174T cells treated 48 h by solvent (0.1% DMSO) or the indicated concentration of ligand. Results were obtained from three separate experiments performed in duplicates. Data are expressed as mean (±s.e.m.) compared with DMSO treated cells, ***P<0.001 **P<0.01 *P<0.05 (Student's *t*-test) compared to LS-CTRL cells. (**e**) RT-qPCR analysis of CYP3A4 mRNA expression in primary cultures of human hepatocytes (three independent donors: HH399, HH404 and HH408) treated 48 h by solvent (0.1% DMSO) or the indicated concentration of ligand. Results were obtained from experiments performed in triplicates. Data are expressed as mean (±s.d.) compared to DMSO treated cells, *P<0.05 (Student's *t*-test). (**f**) Quantifications of CYP3A4 and GAPDH protein expression by western-blot (single experiment, upper panel) and CYP3A4 enzymatic activity (lower panel) in primary culture of human hepatocytes (HH408) treated 72 h by solvent (0.1% DMSO) or 1 μM ligand. Results for enzymatic activity were obtained from one experiment performed in triplicates. Data are expressed as CYP3A4/CellTiter Glow activities ratio as mean (±s.d.), **P<0.005 (Student's *t*-test) compared with DMSO treated cells.

Using medium-throughput ligand screening and a mammalian (HeLa) cell-based activation assay (HG5LN GAL4-PXR-LBD reporter cell line)^14^,^15^, we monitored the agonistic potential of 40 chemicals either alone or in binary mixtures (Supplementary Table 1). Most combinations exhibited additive effects, inducing 50–60% of the transactivation seen with the cholesterol-lowering drug SR12813 (Fig. 1), a potent and well-characterized PXR full agonist^16^,^17^,^18^ (EC_50_ in the 100–200 nM range) used as a reference in all our experiments. However, we observed that the combined use of the organochlorine pesticide TNC and the synthetic oestrogen 17α-ethinylestradiol (EE2), the active component of contraceptive pills, produced more than an additive effect with an induction level of 90% (Fig. 1 and Supplementary Fig. 1). This preliminary observation was confirmed by dose–response experiments. The combination of TNC and EE2 led to a shift of the corresponding activation curve by one order of magnitude towards the low concentrations (Fig. 2a). We next examined the effect of TNC, EE2, and their combination in human liver (HepG2) and colon (LS174T) carcinoma cell lines containing the full-length PXR and the *CYP3A4*-XREM luciferase reporter plasmids. Again, we found that co-treatment yielded to much stronger activation of PXR as monitored by the transactivation of the *CYP3A4* reporter gene (Fig. 2b,c). When used simultaneously, the two compounds activated PXR in a synergistic fashion as illustrated by the theoretical activation curve obtained for the additive combination of EE2 and TNC activities (Fig. 2a–c, red dashed lines calculated using the Bliss independence model^19^). Note that synergism was also observed with other steroidal and organochlorine compound combinations. Supplementary Fig. 2 shows two representative examples associating either EE2 and *cis*-chlordane, or TNC with the natural hormone 17β-estradiol (Fig. 1).

### Cocktail effect on CYP3A4 expression and activity

Consistent with the reporter gene assays, the effectiveness of the individual compounds at increasing the level of endogenous CYP3A4 mRNA was drastically enhanced by the addition of the second compound in LS174T cells expressing PXR but not in control cells (Fig. 2d). We then compared the ability of EE2 and TNC, alone or in combination, to increase *CYP3A4* gene expression in freshly isolated primary human hepatocytes (PHHs) in culture, the most biologically relevant model regarding PXR function. As shown in Fig. 2e, the CYP3A4 mRNA expression was considerably augmented when both EE2 and TNC were used. Accordingly, the enhanced induction of the CYP3A4 protein by the binary mixture (Fig. 2f, upper panel and Supplementary Fig. 3) closely correlated with higher CYP3A4 enzymatic activity (Fig. 2f, lower panel).

As a whole, cell-based assays clearly show that EE2 and TNC act as poor PXR agonists when used separately whereas their combination triggers PXR activation nearly as efficiently as the reference agonist SR12813. Notably, synergism could be observed in various cellular contexts and with different compound combinations, including the natural hormone 17β-estradiol.

### Coactivator recruitment by PXR upon co-treatment

**Figure 3:**
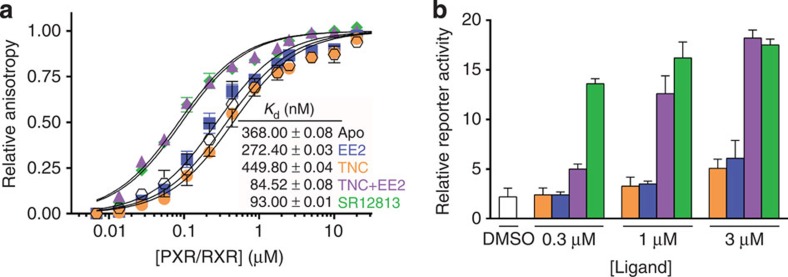
Coactivator recruitment by PXR upon co-treatment. (**a**) Fluorescence anisotropy data showing the relative affinity of the fluorescein-labelled SRC-1 NID for PXR/RXR LBDs heterodimer (20 μM) in the presence of saturating concentrations (60 μM) of TNC, EE2, alone or in mixture, or the PXR agonist SR12813. Assays were performed in three independent experiments and data are expressed as mean (±s.e.m.). (**b**) Mammalian two-hybrid experiment. Gal4-hPXR and VP16-TIF2 interaction monitored in U2OS cells in presence of DMSO, SR12813, EE2, TNC or EE2+TNC. Assays were performed in duplicate in three independent experiments and data are expressed as mean (±s.e.m.).

In order to decipher the molecular mechanism involved in the synergistic activation of PXR-mediated transcription by EE2 and TNC, we characterized their impact on coactivator recruitment. For this purpose, we used fluorescence anisotropy assays with the purified PXR/RXR LBD heterodimers and the fluorescein-labelled NR interaction domain (NID) of SRC-1. We found that, as expected, the PXR agonist SR12813 efficiently enhanced SRC-1 recruitment (Fig. 3a). Interestingly, EE2 and TNC had modest effects on their own but their combination produced a strong increase in the coactivator recruitment, similar to that observed with SR12813. Comparable results were obtained from mammalian two-hybrid experiments (Fig. 3b), suggesting that the active form of the receptor is highly stabilized in presence of both ligands.

### Simultaneous binding of EE2 and TNC to PXR

**Figure 4:**
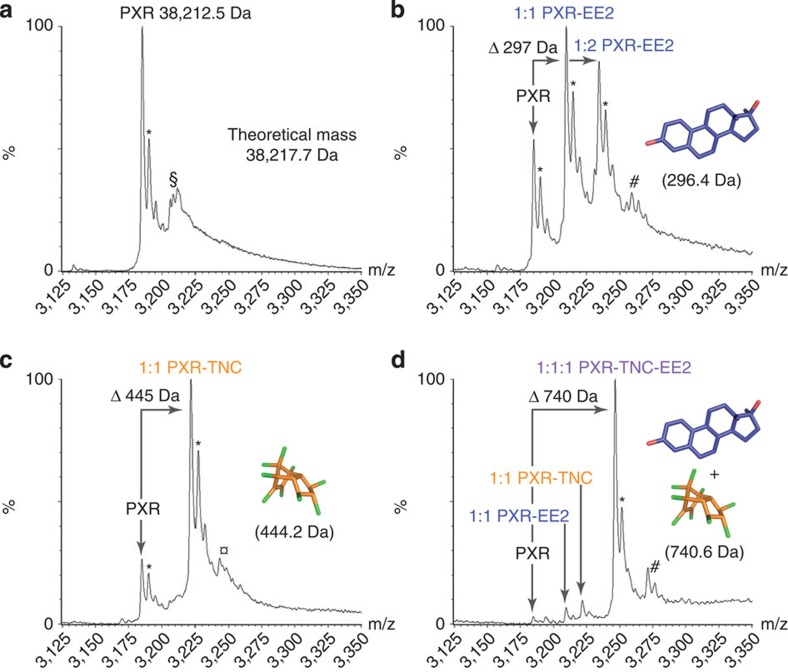
Simultaneous binding of EE2 and TNC to PXR. Mass spectrometry analysis. Non-denaturing ESI-MS was used to characterize PXR LBD (10 μM) in (**a**) the unliganded form or in the presence of (**b**) EE2 (20 μM), (**c**) TNC (20 μM), or (**d**) a mixture of EE2 (20 μM) and TNC (20 μM). *, acetate adducts; $, fortuitous binders 254-324 Da; , fortuitous binder 254 Da; #, non-specific EE2 adducts.

To characterize further the interaction of PXR with EE2 and TNC, we used electrospray ionization mass spectrometry (ESI-MS) under native conditions. Analysed separately, TNC and EE2 were shown to interact with PXR with affinities in the low micromolar range; about 80 and 70% complex were detected when PXR (10 μM) was incubated with 2 molar equivalent excess of TNC or EE2, respectively (Fig. 4a-c). TNC bound to PXR in a 1:1 molar ratio, while EE2 was found to interact with the receptor with 1:1 and 1:2 binding stoichiometries. Binding specificity of the two compounds for PXR ligand-binding pocket was assessed using competition experiments against SR12813. As shown in Supplementary Fig. 4, SR12813 was able to compete efficiently for EE2 and TNC. No non-specific binding was detected with TNC, whereas some residual EE2 was observed in presence of SR12813, suggesting that part of the secondary EE2 binding sites could reside outside the PXR LBP. Analysis of PXR after incubation with the binary cocktail showed a large predominance of a ternary complex corresponding to PXR interacting with EE2 and TNC in a 1:1:1 molar ratio; the global range of affinity of the two compounds binding to PXR was sub-micromolar (Fig. 4d). Together, these data showed that PXR can accommodate EE2 and TNC simultaneously and suggested greater binding affinity of the binary mixture compared to individual compounds.

### EE2 and TNC bind cooperatively to PXR

**Figure 5:**
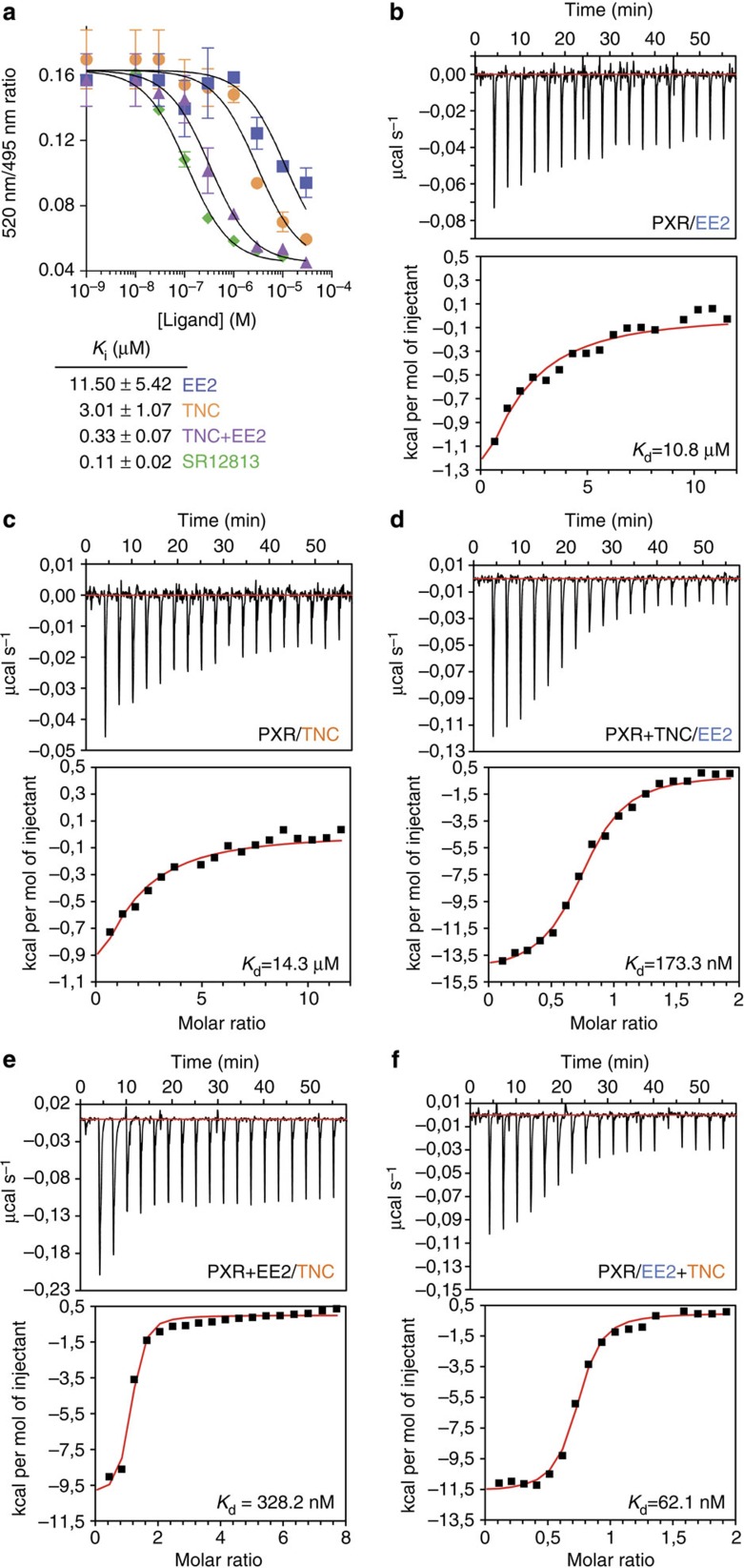
EE2 and TNC bind cooperatively to PXR. (**a**) Inhibition of FRET between fluorescein-labelled PXR ligand and recombinant GST-PXR by SR12813, TNC and EE2, alone or in combination. Results are expressed as the signal from the fluorescein emission divided by the terbium signal to provide a TR-FRET emission ratio. Data are the mean (±s.e.m.) from three separate experiments. (**b-f**) Isothermal titration calorimetry (ITC) characterization of PXR interaction with EE2 and TNC. Ligands were titrated either independently (**b**,**c**), after pre-incubating the receptor with EE2 (**d**) or TNC (**e**), or simultaneously (**f**). In **b**–**f**, representative thermograms (upper row) and corresponding binding isotherms (lower row) are shown. *K*_d_ values are expressed as the mean of two independent experiments.

We next assessed the binding characteristics of EE2 and TNC, alone or in mixture, to PXR. Competitive binding assays using time resolved fluorescence resonance energy transfer between a fluorescent PXR ligand and purified human PXR-LBD (LanthaScreen TR-FRET PXR Competitive Binding Assay) showed that the binary mixture binds 10- to 30-fold more avidly to PXR than TNC and EE2 alone (Fig. 5a). To define the receptor–ligand interactions by a direct method, we next measured the binding affinity constants of various EE2 and TNC combinations by isothermal titration calorimetry (ITC). Before ITC experiments, dose-dependent aggregation of the compounds in working buffer was assessed using dynamic light scattering. Critical aggregation concentration (CAC) was estimated to be in the 50-100 μM range when compounds were diluted serially from 500 μM down to 3 μM in Tris-HCl 20 mM, pH 8.5, NaCl 200 mM, TCEP 1 mM, Tween20 0.05%, DMSO 5%. Accordingly, we cannot rule out that when used at 200-300 μM in some experimental conditions, a fraction of the compounds undergo aggregation despite the dilution process occurring during titration experiments (see Methods). Nevertheless, ITC analyses clearly converged towards similar conclusion of cooperative binding of EE2 and TNC to PXR resulting in increased global binding affinity. ITC data showed much better affinities of EE2 and TNC for PXR when the receptor was pre-incubated with TNC or EE2, respectively (*K*_d_ values in the high-nanomolar range compared to the mid-micromolar range interaction of the compounds tested independently; Fig. 5b-e). Simultaneous titration of EE2 and TNC against PXR was associated with a binding affinity of the binary mixture that was substantially higher than those of the compounds tested alone (Fig. 5f), corroborating the observations derived from ESI-MS and LanthaScreen experiments.

Taken altogether, these *in vitro* data performed with purified material strongly support the notion that both the cooperative binding and synergism observed with EE2 and TNC rely on direct interactions with PXR and not on other cellular mechanisms such as cellular influx/efflux, metabolism, or binding to other cellular targets.

### Structural basis for supramolecular ligand activity

**Figure 6:**
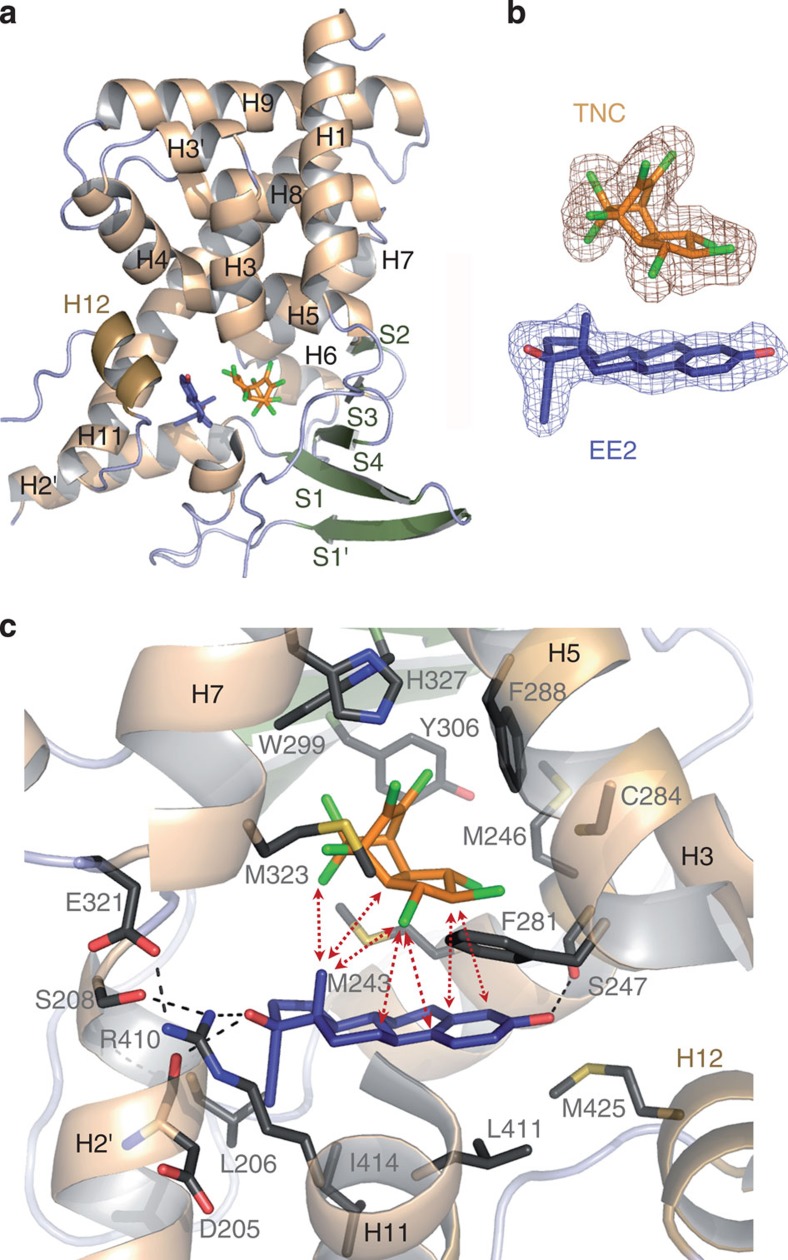
Structural basis for supramolecular ligand activity. (**a**) Overall structure of PXR-LBD in complex with EE2 (blue) and TNC (orange); the structure shows the LBP bordered by helix H12 (light brown) on one side and the β-sheet (green) on the other side; for clarity helix H3 is partially cut. (**b**) Electron density of ligands (2*F*_o_-*F*_c_ map contoured at 1σ). (**c**) Interaction network of ligands with residues of the LBP (grey); red dashed lines highlight the interactions between EE2 and TNC resulting in a mutual stabilization of the compounds in the LBP. Colour code: red, oxygen; blue, nitrogen; yellow, sulphur; green, chlorine; black dashed lines, hydrogen bonds.

To gain structure-based insight into the binding mode of EE2 and TNC to PXR, we solved the crystal structures of PXR-LBD in complex with EE2, TNC, or both at 2.00 Å, 2.55 Å, and 2.25 Å resolution, respectively (Table 1 and Supplementary Fig. 5). In all cases, the PXR-LBD adopts the canonical active conformation, with the C-terminal activation helix H12 capping the LBP (Fig. 6a and Supplementary Fig. 6a,b). Whereas EE2 could be precisely placed in the electron densities obtained for both the PXR–EE2 and PXR–TNC–EE2 complexes, TNC could be positioned unambiguously in the ternary complex only (Fig. 6b and Supplementary Fig. 6c,d). The poorly defined electron density of TNC in the PXR–TNC complex likely reflects high mobility of this ligand in the pocket. In contrast to EE2 which is engaged in several hydrogen bonds maintaining the ligand in a defined location, TNC has no polar group and likely adopts an ensemble of different positions and/or orientations in the LBP.

EE2 binds closely adjacent to H12 with a binding mode that is reminiscent of that seen for 17β-estradiol^20^ (Supplementary Fig. 7a), both in presence or absence of TNC. The 3-hydroxyl group on the A-ring of EE2 forms an hydrogen bond with S247 (helix H3), whereas the 17β-hydroxyl group on the D-ring is hydrogen bonded with R410 in helix H11 and the main chain oxygen atom of D205 from helix H2' (Fig. 6c). The position of R410 is further stabilized by a network of hydrogen bonds involving S208 (helix H2') and E321 from the loop preceding H7. The remaining contacts between EE2 and the protein involve van der Waals interactions with several hydrophobic residues. The particular position of EE2 in a restricted region of PXR LBP leaves a significant portion of the pocket unoccupied and available for additional interactions (Supplementary Fig. 6a,c). As seen in the ternary complex, this empty region can accommodate one molecule of TNC which is stabilized in a well-defined position *via* a number of interactions with both EE2 and the protein. As illustrated in Fig. 6c, eight van der Waals contacts of 3.7 to 4.5 Å in length could be measured between EE2 and TNC. These inter-ligand contacts generate a mutual stabilization of the compounds in the LBP and account for the enhanced binding affinity of the binary mixture (Fig. 5). On the protein side, TNC forms essentially nonpolar interactions with ten residues, including F281, F288, W299, Y306, M323 (Fig. 6c) and two weak halogen bonds with Q285 and C284 from helix H5.

In keeping with the robust agonistic activity of the binary mixture, superposition of the supramolecular ligand-bound structure with that of PXR in complex with SR12813 (ref. 21) or rifampicin^22^ reveals that the binding sites of EE2 and TNC overlap those of the two PXR agonists (Supplementary Fig. 7b,c). Hence, the molecular assembly of EE2 and TNC into the LBP of PXR can be regarded as a supramolecular ligand whose functional properties rely on intermolecular interactions and differ from those of the individual components.

## Discussion

Most current knowledge of EDCs action is derived from data sets that use single molecule exposure, with few studies taking into account the more realistic situation where humans are simultaneously and chronically exposed to low doses of multiple EDCs. Indeed, a growing number of studies indicate that human risk assessment approaches based on single molecule exposure underestimate the risk for adverse effects of chemicals^10^. The evaluation of mixture effects by regulatory bodies has been mainly hampered by the huge numbers of pollutants and potential combinations, but also by the lack of knowledge of the molecular pathways involved. This study provides both the first detailed mechanistic explanation and a proof of concept for the synergistic action of two compounds *via* their simultaneous interaction with the LBP of a NR. Our results provide not only new insight as to how low doses of EDCs or drugs may affect physiology and homeostasis, but also suggest that the concomitant binding of chemicals stabilizing each other in NR LBPs likely corresponds to one of the possible mechanisms accounting for the cocktail effect by which compounds toxicity is exacerbated.

Reports describing the simultaneous binding of two or more compounds to a common protein binding site are very few in number. One of the rare examples is that of the *Staphylococcus aureus* multidrug-binding transcription repressor QacR which was shown to bind concomitantly to ethidium and proflavin. However no cooperative binding mechanism was observed in this case^23^. Other examples include the cytochrome CYP3A4 bound to two molecules of ketoconazole^24^ or the peroxisome proliferator activated receptor gamma (PPARγ) which can accommodate two copies of FMOC-L-Leucine^25^, or endogenous fatty acids^26^. However, in both cases the bound molecules were of the same type and the possibility of synergism between the two ligands was not addressed. Besides the interaction of several compounds with a unique binding site, two studies reported on the oestrogen antagonist 4-hydroxytamoxifen and a synthetic ligand binding to two alternate sites of the oestrogen receptor β and PPARγ LBDs, respectively^27^,^28^. Again, no cooperativity was reported between these dual binding sites. Our study shows that individually, EE2 and TNC are too small to make all the necessary interactions ensuring high binding affinity and effective stabilization of the active conformation of the receptor. In contrast, when associated in a binary mixture, EE2 and TNC form a supramolecular ligand that fills a larger fraction of the PXR LBP, and displays apparent functional properties (*e.g*. activity and binding affinity) comparable to those of the full PXR agonists, SR12813 and rifampicin.

Cloning of PXR orthologues from human, rabbit, rat and mouse has shown that the ligand-binding domain has diverged considerably between the different species, leading to specific ligand-binding and activation profiles^29^,^30^. This species-specific induction pattern of PXR is possibly an adaptive response to the environment and a need to adjust toxicological responses to endogenously produced substances. Therefore, additional studies should be performed to test the potential EE2/TNC synergy *in vivo* in these species. Notably future studies will be completed in mice, along with the use of PXR-knock-out models and long term exposures with these compounds, to confirm the role of PXR and the physiological relevance of the EE2/TNC synergism on drug and bile acid metabolism, hepatic steatosis, or liver regeneration for instance.

Large LBPs such as those of PXR or PPARγ are obviously predisposed to accommodate several molecules at the same time. However, in contrast to the perception that most NRs possess a well-defined pocket to account for the specific binding of a unique endogenous ligand, several structural studies have revealed that their LBPs exhibit a greater conformational flexibility than previously thought. Importantly, this structural adaptability allows the oestrogen (ERα)^31^, thyroid (TRβ)^32^, or glucocorticoid (GR)^33^ receptors to expand their binding pocket and accommodate much larger ligands^34^. Another interesting example is the oestrogen-related receptor gamma (ERRγ) whose LBP appears too small to bind any ligand, yet can expand it to accommodate rather large compounds^35^. Considering the huge chemical and size diversity of xenobiotics and the high structural plasticity of NR LBPs, the mechanism defined for PXR is likely to occur also with other members of the NR superfamily, with broad reaching implications in the fields of endocrine disruption, chemicals risk assessment and toxicology. The development of novel supramolecular chemistry-based therapeutic options could also benefit from the discovery reported here.

## Methods

### Ligands

TNC, EE2, E2 and CC were purchased from Sigma-Aldrich. SR12813 was purchased from Tocris Bioscience. The provenance of compounds used for medium-throughput screening is described in Supplementary Table 1. All compound stock solutions were prepared at 10 mM in DMSO.

### Plasmids

The [−7.8/−7.2] XREM-CYP3A4 (XREM-CYP3A4 pGL3b) luciferase plasmid has been described previously^36^. The 17Mx5-Glob-LUC containing five GAL4 binding sites upstream of the luciferase reporter gene and the VP16 (activation domain)-TIF2 (amino acids 624-869 containing three L*XX*LL motifs) are gifts from Hinrich Gronemeyer (IGBMC, Illkirch, France). The pM-hPXR expression vector was generated by inserting a PCR fragment corresponding to the full-length hPXR in the pM vector (CLONTECH).

### Cell lines

LS174T stable human PXR transfectant (LS-PXR2) and the corresponding control cells (LS-CTRL), HEPG2-PXR and HG5LN GAL4-PXR reporter cell lines were previously described^17^,^37^,^38^. Briefly, LS-PXR2 was obtained after stable transfection of the LS174T cells with the pcDNA3.1-hPXR (residues 1-434)-neomycin expressing plasmid. HEPG2-PXR was obtained after stable transfection of the same PXR expressing plasmid and the XREM-CYP3A4 pGL3b reporter plasmid^38^. HG5LN GAL4-PXR reporter cell line was established by stable transfection of pSG5-GAL4DBD (residues 1-147)-hPXR LBD (residues 107-434)-puromycin and GAL4-RE5-βGlobin-luciferase-neomycin plasmids^17^. Finally, LS174T-PXR reporter cell line was established by stable transfection of the CYP3A4-luciferase-hygromycine plasmid in the LS-PXR2 cell line. All cell lines were grown in DMEM medium (Invitrogen) supplemented with 10% fetal calf serum, L-glutamine, and antibiotics (Invitrogen).

### Medium-throughput screening

HG5LN GAL4-PXR reporter cell lines (100 μl) were seeded at a density of 25,000 cells per well in 96-well white opaque tissue culture plates (Greiner CellStar). 24 h later, negative (DMSO 0,1%) and positive (SR12813 3 μM) controls, and the 40 tested compounds (50 μl) were added into the wells as indicated in Supplementary Table 1. Then the ligand to be tested in combination with the different ligands was added (50 μl). Cells were incubated at 37 °C for 16 h. At the end of the incubation period, culture medium was replaced with medium containing 3.10^−4^ M luciferin. Luciferase activity was measured for 2 s in intact living cells using a plate reader (PerkinElmer Luminometer).

### Transactivation assays

HG5LN GAL4-PXR, HEPG2- and LS174T-PXR reporter cell lines were seeded at a density of 25,000 cells per well in 96-well white opaque tissue culture plates (Greiner CellStar). Compounds to be tested were added 24 h later, and cells were incubated at 37 °C for 16 h. At the end of the incubation period, culture medium was replaced with medium containing 3.10^−4^ M luciferin. Luciferase activity was measured for 2 s in intact living cells using a plate reader (PerkinElmer Luminometer). EC_50_ values were measured using GraphPad Prism (GraphPad Software Inc.).

### Preparation of primary human hepatocytes

Liver samples were obtained from liver resections performed in adult patients for medical reasons unrelated to our research program or from donors when the liver was considered unsuitable for organ transplantation. The use of human specimens for scientific purposes was approved by the French National Ethics Committee. Written or oral informed consent was obtained from each patient or family prior to surgery. The clinical characteristics of the liver donors are presented in Supplementary Table 2. Hepatocytes were isolated by using a two-step perfusion protocol and cultured as described previously^39^. Briefly, several veins apparent on the cut edge of the lobectomy were used for sequential perfusions with a washing buffer (10 mM HEPES, 136 mM NaCl, 5 mM KCl, 0.5% glucose, pH 7.6), with a calcium chelating buffer (washing buffer complemented with 0.5 mM EGTA), with the washing buffer and then with a collagenase IV solution (washing buffer supplemented with collagenase IV, 200 U ml^−1^, Sigma) for 20 min. After gentle disruption of the tissue and filtration through a 250 μm mesh, the post-collagenase homogenate was centrifuged at low speed (50 g for 5 min) to pellet the hepatocytes. Hepatocytes were seeded in collagen type-I coated dishes (Becton Dickinson) at 1.7 10^5^ cells per cm^2^ in a hormonally and chemically defined medium for short term culture consisting of a mixture of William's E and Ham's F-12 (1:1 in volume) and additives as previously described^40^ supplemented with 2% heat inactivated fetal calf serum (FCS). After overnight attachment, the medium and unattached cells were removed and fresh medium without FCS was added. Hepatocytes were treated with the molecules at day 2 post-seeding for 24 or 48 h.

### Reverse transcription-PCR and real-time quantitative PCR

Total RNA was extracted from 200,000 cells using the RNeasy mini kit (Qiagen) or Trizol reagent (Invitrogen) and treated with DNAse-1. The first strand cDNA was synthetized using Superscript II (Invitrogen) or MMLV (Invitrogen) and random hexamers. Primer sequences are provided in Supplementary Table 3. Gene expression was normalized with GAPDH and the level of expression was compared with the mean level of the corresponding gene expression in untreated cells and expressed as n-fold ratio. The relative amount of RNA was calculated with the 2^ΔΔ^CT method.

### Western-immunoblotting analysis

Total protein extracts were prepared from 500,000 cells with RIPA buffer (Tebu-Bio) in presence of antiproteases (Roche). The protein concentration was determined by the bicinchoninic acid method, according to the manufacturer's instructions (Pierce Chemical Co.). Bovine serum albumin (Pierce Chemical Co.) was used as standard. Cell lysates were resolved on SDS-PAGE and transferred to a Hybond-ECL membrane (GE Healthcare). Membranes were incubated with rabbit anti-CYP3A4 (5316, 1/10,000, Epitomics) or mouse anti-GAPDH (sc#32233, 1/5,000, Santa Cruz) monoclonal antibodies. Immunocomplexes were detected with horseradish peroxidase-conjugated rabbit (A0545, 1/10,000, Sigma) or mouse (A9044, 1/10,000, Sigma) secondary antibodies followed by enhanced chemiluminescence reaction (Millipore). Chemiluminescence was monitored using a ChemiDoc-XRS^+^ apparatus (Bio-Rad Laboratories).

### Measurement of CYP3A4 activity

Primary human hepatocytes were seeded in 96-well plates and treated with the test compounds or vehicle for 48 h. CYP3A4 activity was detected using the P450-Glo CYP3A4 luciferin-IPA Enzyme Activity Kit (Promega) according to the manufacturer's instructions. Cell number was normalized using CellTiter-Glo Luminescent Cell Viability Assay (Promega).

### Statistical analyses

For the analysis of the correlation between parametric data, Stutent's *t*-test was used, while the Mann–Whitney *U*-test was used for nonparametric data. Differences were considered statistically significant when *P*-values were ***P<0.001 **P<0.01 *P<0.05.

### Preparation of PXR/RXR for fluorescence anisotropy assays

The human PXR-LBD (130-434; pDB-His-MBP vector) and the human RXR-LBD (223-462; pET-3a vector) were overproduced in *Escherichia coli* BL21(DE3). Cells were grown at 37 °C in LB medium supplemented with the appropriate antibiotic until OD_600_ reached 0.6. Expression of T7 polymerase was induced by addition of isopropyl-β-D-thiogalactoside to a final concentration of 0.5 mM. After 16 h of incubation at 20 °C, cell cultures were harvested by centrifugation at 6,000*g* for 15 min. Cell pellets from 3 L of His_6_-MBP-PXR-LBD culture and from 1 l of non-tagged RXR-LBD culture were resuspended in 150 ml buffer A (20 mM HEPES pH 7.5, 250 mM NaCl, 15 mM imidazole, 5% (v/v) glycerol) supplemented with a protease inhibitor cocktail tablet (cOmplete, EDTA-free, Roche). The suspension was then lysed by sonication and centrifuged at 18,000*g* at 4 °C for 30 min. The supernatant was loaded onto a Ni^2+^-affinity column (HisTrap 5 ml; GE Healthcare) equilibrated with buffer A, using the ÄKTA pure system (GE Healthcare). The column was washed with 20 volumes of buffer A and 20 volumes of buffer A containing 50 mM imidazole. The His_6_-MBP-PXR/RXR heterodimer was eluted with buffer A containing 100 mM imidazole. The fractions containing the heterodimer were pooled and incubated for 48 h at 4 °C with the tobacco etch virus protease to cleave the His_6_-MBP tag, and then further reloaded onto the Ni^2+^-affinity column to eliminate the tag. The flow-through containing the PXR/RXR heterodimer was then purified using a gel filtration column (Superdex 200 16/60; GE Healthcare) equilibrated with buffer B (10 mM Tris-HCl pH 7.5, 250 mM NaCl, 1 mM DTT and 5% (v/v) glycerol). The fluorescein-labelled SRC-1 fragment (residues 570–780) was prepared using the protocol previously described^41^. Briefly, the SRC-1fragment was expressed in *E. coli* BL21(DE3) as a fusion with an inducible self-splicing intein (Sce VMA) and a chitin-binding domain using the vector pTYB1 (New England Biolabs). The cells were grown at 37 °C to 0.6 at OD_600_ and then at 17 °C overnight. The fusion protein was purified using chitin resin (New England Biolabs). Intein cleavage was induced using 2 mM cys-fluor with 50 mM MESNA, releasing C-terminally labelled SRC-1. Excess cys-fluor was removed using a phenyl sepharose resin (Amersham). SRC-1 was further purified using size-exclusion chromatography. Thin-layer chromatography was used to confirm that there was no free fluorescein label in the purified samples.

### Steady-state fluorescence anisotropy

Measure of the binding affinities of the coactivator fragment for the PXR/RXR heterodimer in the absence and presence of various ligands was performed using a Safire2 microplate reader (TECAN). The excitation wavelength was set at 470 nm and emission measured at 530 nm for the fluorescein-tagged fragment. Assays were carried out in the following buffer solution 20 mM Tris-HCl, pH 7.5, 150 mM NaCl, 1 mM TCEP and 5% (v/v) glycerol. We initiated the measurements at the highest concentration of protein (20 μM) and diluted the protein sample twofold successively with the buffer solution. For each point of the titration curve, the protein sample was mixed with 5 nM of fluorescent fragment and a 3 molar excess of ligand (60 μM final concentrations). Binding data were fitted using a sigmoidal dose–response model using GraphPad Prism (GraphPad Software Inc.).

### Mammalian two-hybrid experiments

Gal4-hPXR and VP16-TIF2 interaction was monitored on 17Mx5-Glob-LUC reporter construct. Transient transfections assays were performed in U2OS cells using Jet-PEI (Ozyme) according to manufacturer's instructions. Luciferase assays were performed with the Promega dual-reporter kit, according to the manufacturer's instructions. *Renilla* luciferase encoded by the normalization vector phRLTK (Promega) was used as internal control for firefly luciferase normalization.

### Mass spectrometry

Mass spectrometry experiments were carried out on an electrospray time-of-flight mass spectrometer (LCT, Waters) equipped with an automated chip-based nanoESI device (Triversa Nanomate, Advion Biosciences). External calibration was done in the positive ion mode over the mass range *m/z* 500–5,000 using the multiply charged ions produced by 0.5 μM horse heart myoglobin solution diluted in water/acetonitrile 50/50 mixture acidified with 0.5% (v/v) formic acid. Purified PXR(130-434)-SRC1 was buffer exchanged against 50 mM ammonium acetate (NH4Ac), pH 8.0 using NAP5 desalting columns (illustra NAP-5 Columns, GE Healthcare Life Sciences). Protein concentration was determined spectrophotometrically (*ɛ*_280 nm_=26,210 l mol^−1^ cm^−1^). Analysis of EE2 and TNC binding to PXR(130-434)-SRC1 was achieved in 50 mM NH_4_Ac pH 8.0 keeping a constant 5% amount of isopropanol (v/v). Protein concentration was set to 10 μM and different compound concentrations ranging from 20 to 80 μM were tested. Incubations lasted 5 min at 18 °C. Mass spectra were recorded using low cone voltage (*V*_c_, 20 V) and elevated interface pressure (Pi, 5 mbar).

### Lanthascreen TR-FRET PXR competitive binding assay

GST-hPXR-LBD (10 nM) was incubated with different concentrations (10–30 μM) of TNC, EE2, TNC and EE2, and SR12813 in the presence of Fluormone PXR ligand (40 nM) and Lanthascreen terbium-anti-GST antibody (10 nM). To read a LanthaScreen TR-FRET assay, the fluorimeter (PHERAstar FS; BMG LABTECH) is configured to excite the terbium donor around 340 nm, and to separately read the terbium emission peak that is centred at ∼490 nm, and the fluorescein emission that is centred at ∼520 nm. Results are expressed as the signal from the fluorescein emission divided by the terbium signal to provide a TR-FRET emission ratio. Fluorescence ratio data were fitted using a sigmoidal dose–response model using GraphPad Prism (GraphPad Software Inc.).

### Isothermal titration calorimetry

Purified PXR(130-434)-SRC-1 was dialysed overnight against Tris-HCl 20 mM, pH 8.5, NaCl 200 mM, TCEP 1 mM using 10 kDa molecular weight cut-off dialysis cassettes (Slide-A-Lyzer 0.5 ml 10 K MWCO, Thermo Scientific). Protein concentration was determined spectrophotometrically (*ɛ*_280 nm_=26,210 l mol^−1^ cm^−1^). Duplicate experiments were performed on Microcal ITC200 (Malvern) operating at 25 °C. Titrations were carried out in Tris-HCl 20 mM, pH 8.5, NaCl 200 mM, TCEP 1 mM supplemented with 0.05% Tween 20 and 5% DMSO (syringe, sample and reference cells). PXR (5 μM) was disposed in 200 μl cell and compounds were delivered from 40 μl syringe. Compound solutions were set to 300 μM when tested individually (Fig. 5b,c), 50 μM each when used simultaneously (Fig. 5f) and 50 μM (EE2, Fig. 5d) or 200 μM (TNC, Fig. 5e) when tested after pre-incubation of PXR with 50 μM TNC or EE2, respectively. Heat exchanges were monitored throughout titrations consisting of 19 injections (one time 0.5 μl followed by 18 times 2 μl) of compound solutions into the cell containing PXR solution. Data analysis and thermodynamic parameter fitting used Microcal Origin software (Malvern).

### Protein production and purification for structural studies

The human PXR-LBD (130-434) was co-produced with a fragment of the steroid receptor coactivator-1 (SRC-1, 623-710) to enhance PXR stability. The PXR-LBD gene was cloned into pRSET-A with a His_6_ tag at the N-terminus (gift from Matthew Redinbo, University of North Carolina at Chapel Hill, USA), and the SRC-1 fragment gene has been inserted into the pACYC184 vector. Proteins were overproduced in *E. coli* BL21(DE3) cells overnight at 20 °C in LB medium without any ligand. After culture cells were harvested by centrifugation and the pellets resuspended in lysis buffer (20 mM Tris pH 7.5, 250 mM NaCl, 5% (v/v) glycerol) supplemented with lysozyme (1 μg ml^−1^) and a protease inhibitor cocktail tablet (cOmplete, EDTA-free, Roche), and then subjected to sonication. The clarified cell lysate was applied onto a Ni^2+^-affinity column (HisTrap 5 ml; GE Healthcare) equilibrated with lysis buffer supplemented with 10 mM imidazole. The eluted PXR-LBD was then applied onto a gel filtration column (Superdex 75 26/60; GE Healthcare) equilibrated with a buffer containing 20 mM Tris pH 7.8, 250 mM NaCl, 5% (v/v) glycerol, 5 mM DTT, 1 mM EDTA. The PXR-LBD was concentrated and stored at −40 °C.

### Crystallization

Prior to crystallization assays the purified PXR-LBD (2.4 mg ml^−1^) was mixed with EE2 (2.5 molar equivalents), TNC (2.5 molar equivalents), or EE2+TNC (2.5 molar equivalents each). Co-crystals with EE2 were obtained in 100 mM NaCl, 100 mM imidazole pH 7.1, 10% (v/v) isopropanol, in 100 mM imidazole pH 7.1, 10% (v/v) isopropanol with TNC alone, and in 50 mM NaCl, 50 mM LiCl, 100 mM imidazole pH 7.1, 10% (v/v) isopropanol with EE2+TNC mixture.

### Data collection and structure determination

For all complexes, native data were collected from one crystal cryoprotected with 20% (v/v) MPD on the ID23-2 beamline at the European Synchrotron Radiation Facilities (*λ*=0.8726 Å, 100 K), Grenoble, France. Data were processed and scaled with XDS and XSCALE^42^. Crystals belong to space group *P* 4_3_2_1_2. The X-ray structures were solved and refined using Phenix (phenix.refine)^43^ and COOT^44^. The percentages of residues located in the favoured Ramachandran plot region are 98.1, 98.2 and 97.9% for the PXR−EE2, PXR−TNC and PXR−EE2−TNC complex structures respectively (calculated with MolProbity^45^). Data collection and refinement statistics are summarized in Table 1. Figures were prepared with PyMOL (http://pymol.org/).

## Additional information

**Accession codes:** The atomic coordinates and structure factors have been deposited in the Protein Data Bank under accession codes 4X1F (EE2), 4XAO (TNC) and 4X1G (EE2+TNC).

**How to cite this article:** Delfosse, V. *et al*. Synergistic activation of human pregnane X receptor by binary cocktails of pharmaceutical and environmental compounds. *Nat. Commun*. 6:8089 doi: 10.1038/ncomms9089 (2015).

## Supplementary Material

Supplementary Information Supplementary Figures 1-7, Supplementary Tables 1-3 and Supplementary References

## Figures and Tables

**Table 1:** Data collection and refinement statistics.

	EE2 (4X1F)	TNC (4XAO)	EE2+TNC (4X1G)
**Data collection**			
Space group	*P* 4_3_ 2_1_ 2	*P* 4_3_ 2_1_ 2	*P* 4_3_ 2_1_ 2
Cell dimensions			
*a*, *b*, *c* (Å)	91.34, 91.34, 85.35	92.30, 92.30, 86.30	91.34, 91.34, 85.49
*α*, *β*, *γ* (°)	90.00, 90.00, 90.00	90.00, 90.00, 90.00	90.00, 90.00, 90.00
Resolution (Å)	45.67–2.00 (2.11–2.00)*	41.28–2.58 (2.69–2.58)*	40.85–2.25 (2.38–2.25)*
*R*_sym_	0.080 (0.489)	0.110 (0.472)	0.091 (0.453)
*I* / σ*I*	20.1 (4.7)	14.7 (4.1)	12.4 (3.1)
Completeness (%)	100.0 (100.0)	96.8 (78.3)	97.7 (99.0)
Redundancy	10.4 (10.4)	7.8 (7.3)	5.4 (5.2)
**Refinement**			
Resolution (Å)	45.67–2.00	41.28–2.58	38.71–2.25
No. reflections	24,998	12,258	17,316
*R*_work_/*R*_free_	0.182/0.209	0.189/0.239	0.174/0.218
No. atoms			
Protein	2,158	2,171	2,208
Ligand/ion	38	12	53
Water	170	67	122
*B*-factors			
Protein	30.19	39.48	34.80
Ligand/ion	40.29	57.14	48.99
Water	37.31	40.64	39.36
R.m.s.deviations			
Bond lengths (Å)	0.009	0.002	0.008
Bond angles (°)	0.996	0.569	1.067

R.m.s, root-mean square; TNC, *trans*-nonachlor. *Values in parentheses are for highest-resolution shell.
