# Supplementary material for: Synergistic activation of human pregnane X receptor by binary cocktails of pharmaceutical and environmental compounds
Source: Nat Commun. 2015 Sep 3;6:8089. doi: 10.1038/ncomms9089 (PMC4569708; doi:10.1038/ncomms9089)
Supplement: Supplementary Information — Supplementary Figures 1-7, Supplementary Tables 1-3 and Supplementary References [file ncomms9089-s1.pdf]

Supplementary Figures

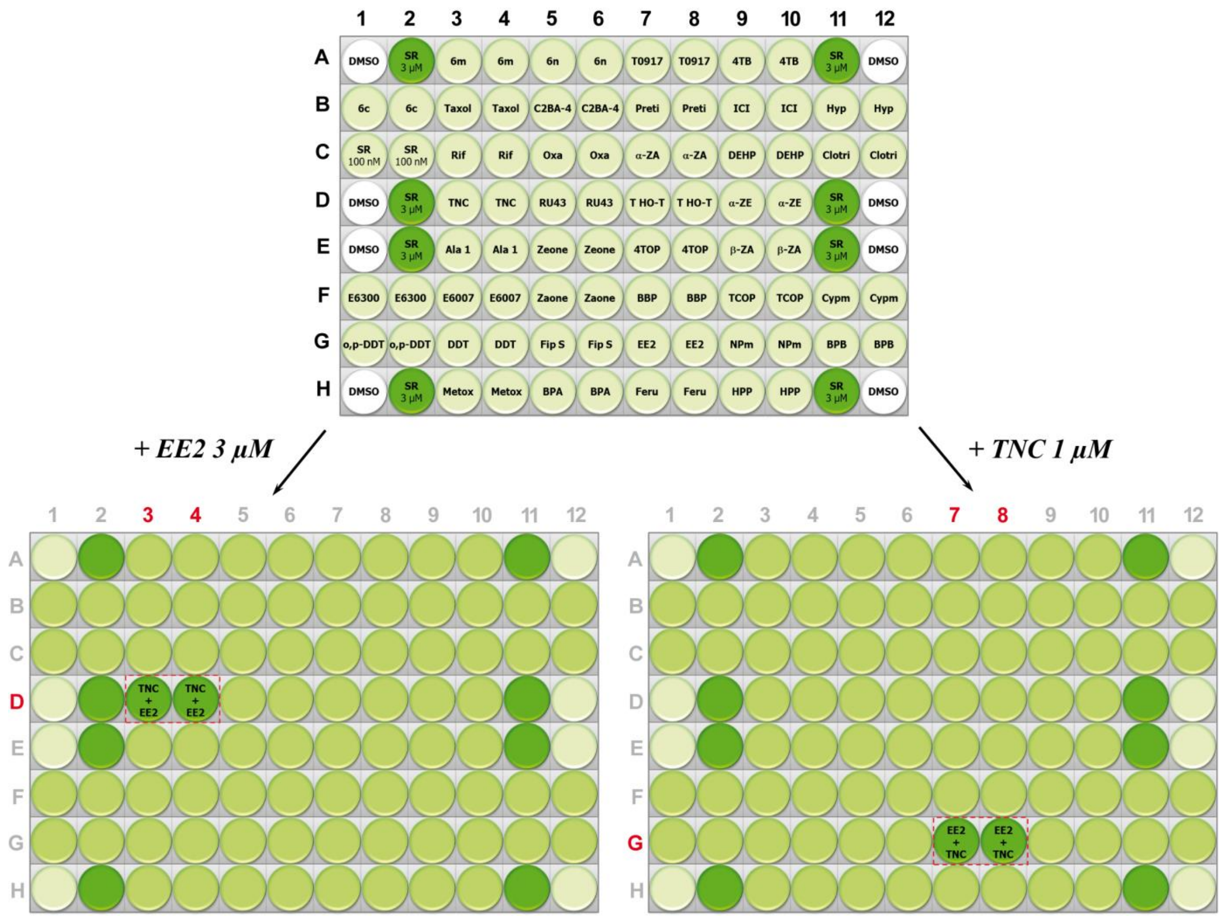

Supplementary Figure 1 | Medium-throughput screening. Schematic results for EE2 and TNC are described. Color code: white, 0-25% activity; light green, 25-50% activity; medium green, 50-75% activity; dark green, 75-100% activity.

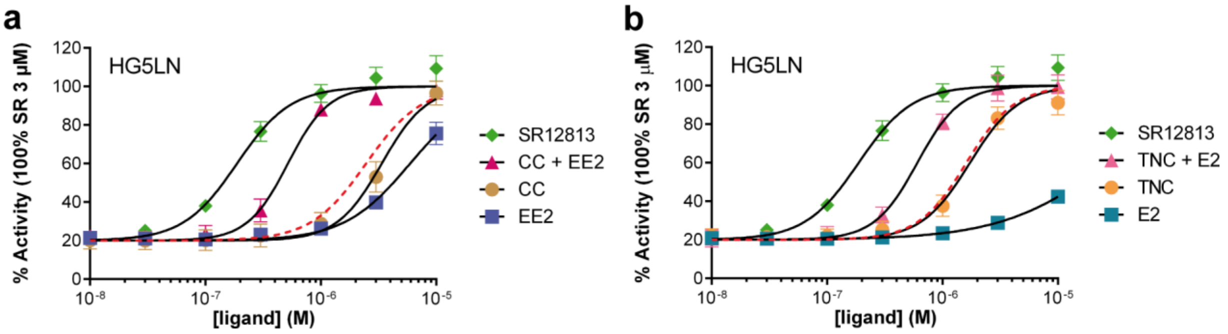

Supplementary Figure 2 | Synergistic activation of PXR by estrogen and pesticide combinations. HG5LN GAL4-PXR-LBD cells were exposed to different concentrations of SR12813, *cis*-chlordane (CC) and EE2 either alone or in combination (**a**), or different concentrations of SR12813, TNC and 17β-estradiol (E2) either alone or in combination (**b**). Red dashed lines represent the theoretical activation curves obtained for the additive combination of estrogens and pesticides activities. Assays were performed in triplicate in three independent experiments and data are expressed as mean (±s.e.m.).

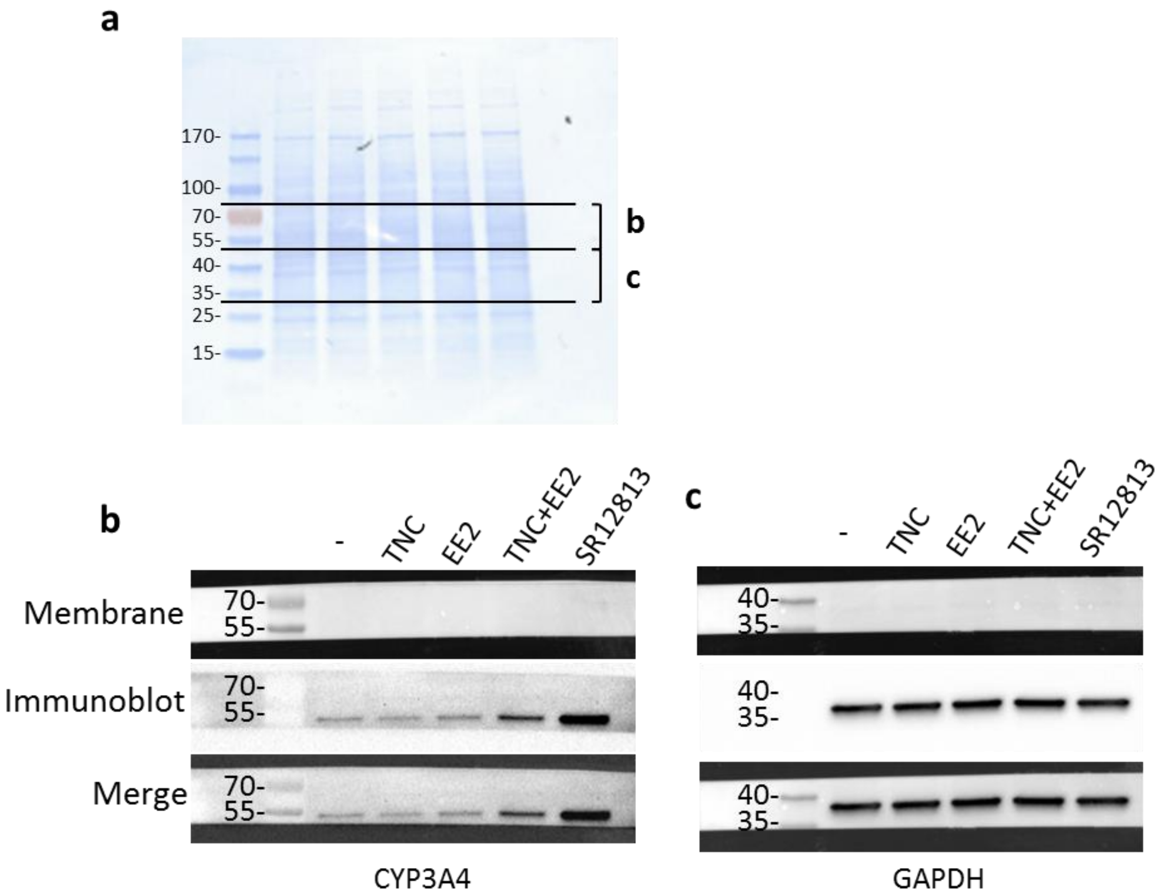

Supplementary Figure 3 | Western-immunoblotting analysis. (**a**) Amido-black staining of the membrane before immunoblotting. Immunoblotting of CYP3A4 (**b**) and GAPDH (**c**) Upper panel: cut up membrane; middle panel: immunoblot; lower panel: merge.

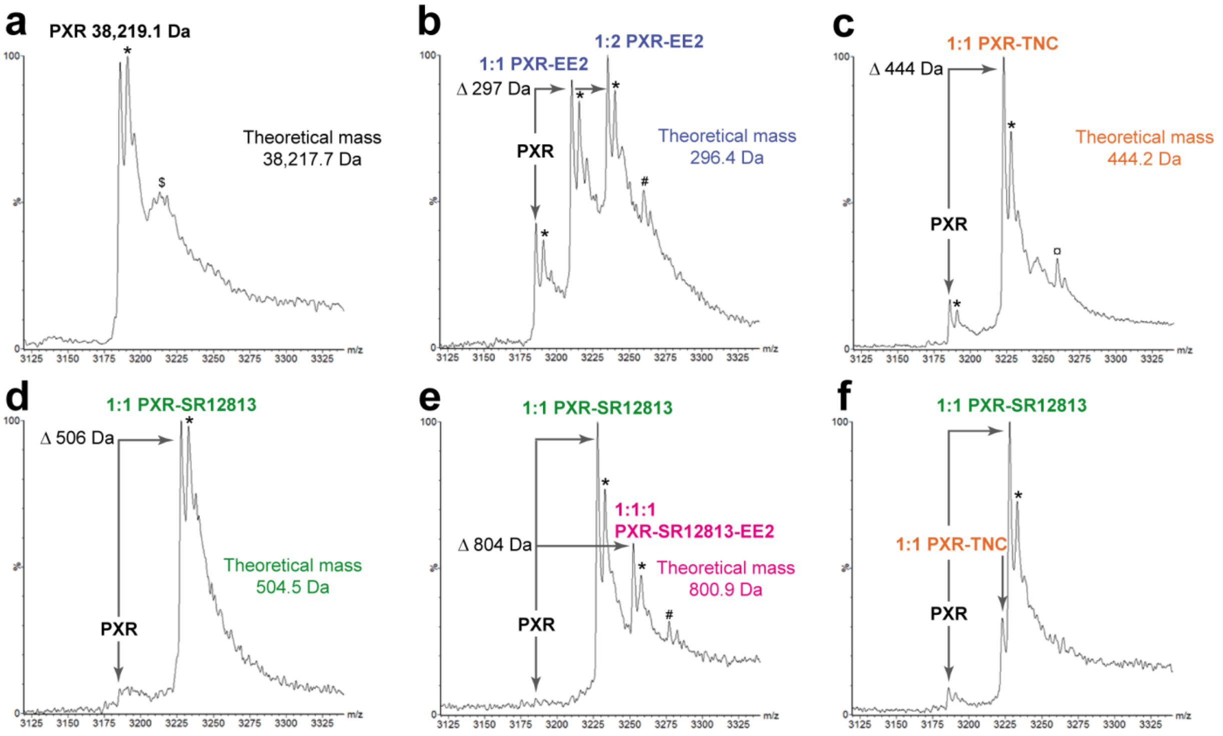

Supplementary Figure 4 | Mass spectrometry analysis. Non-denaturing ESI-MS spectra of unliganded PXR (**a**), PXR–EE2 (**b**), PXR–TNC (**c**), and PXR–SR12813 (**d**) complexes. SR12813 was used to compete with EE2 (**e**) and TNC (**f**) for binding to PXR. *, acetate adducts; $, fortuitous binders 254-324 Da; ¤, fortuitous binder 254 Da; #, non-specific EE2 adducts.

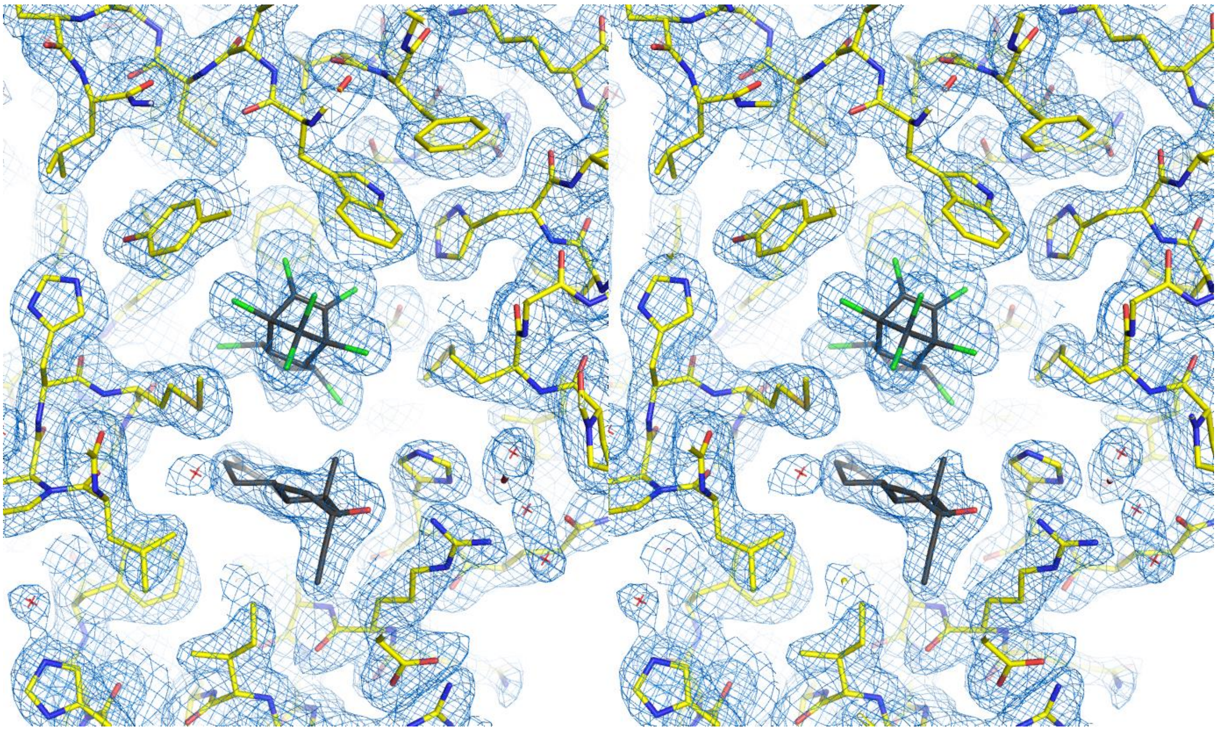

Supplementary Figure 5 | Stereo view of a section of electron density map. Cross-eye stereo views of sections of 2*Fo*-*Fc* map (contoured at 1σ) show clear electron density of PXR from the PXR–TNC–EE2 complex. The corresponding model to this part of the map is shown in stick mode.

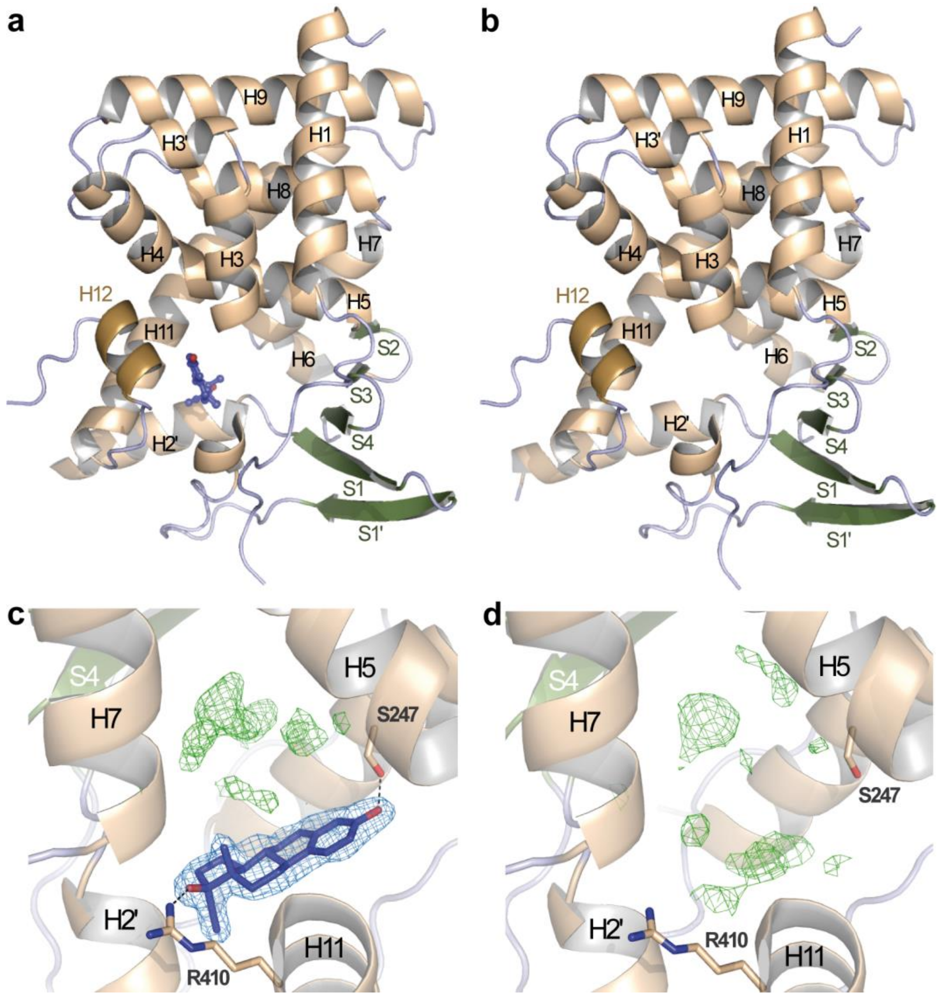

Supplementary Figure 6 | Structures of PXR obtained with EE2 and TNC alone. Overall structure of PXR-LBD in complex with EE2 (blue) (**a**), and crystallized in presence of TNC (**b**); structures show the LBP bordered by helix H12 (light brown) on one side and the β-sheet (green) on the other side; for clarity helix H3 is partially cut. (**c**) Close up view of the LBP of the PXR–EE2 complex structure; one EE2 molecule has been positioned unambiguously (blue; electron density = $2F_o$-F_c map contoured at 1σ); the green electron density (F_o-F_c map contoured at 3σ) could not be assigned unequivocally although mass spectrometry experiments suggested the possibility of two EE2 molecules in the LBP of PXR. (**d**) Close up view of the LBP of PXR crystallized in presence of TNC; the green electron density (F_o-F_c map contoured at 3σ) could not be assigned unequivocally although mass spectrometry experiments suggested the presence of one TNC molecule in the LBP of PXR. Color code: red, oxygen; blue, nitrogen; black dashed lines, hydrogen bonds.

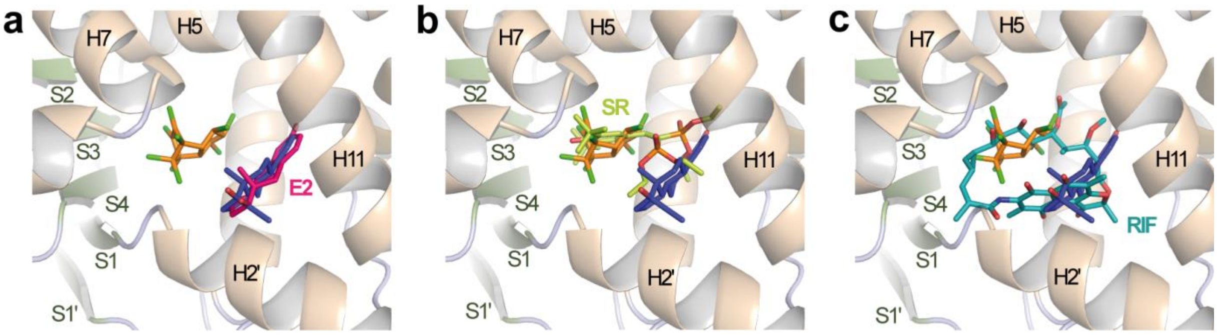

Supplementary Figure 7 | EE2 and TNC together fill the LBP of PXR similarly as full agonists. Structure superposition of PXR–TNC–EE2 complex with PXR–E2 complex[1] (**a**), PXR–SR12813 complex[2] (**b**), and PXR–Rifampicin complex[3] (**c**).

Supplementary Tables

Supplementary Table 1 | Compounds used for screening

Ligand	Well position	Concentration[#]	Ligand	Well position	Concentration[#]
Compound 6m*	A3-A4	1 nM	Alachlor***	E3-E4	1 µM
Compound 6n*	A5-A6	1 nM	Zearalenone***	E5-E6	1 µM
T0901317**	A7-A8	10 nM	4-tert-octylphenol***	E7-E8	1 µM
4-tert-butyl-2-methylphenol***	A9-A10	1 µM	β-zearalanol***	E9-E10	1 µM
Compound 6c*	B1-B2	10 nM	4-methylbenzylidene camphor***	F1-F2	1 µM
TAXOL***	B3-B4	30 nM	Octyl dimethyl PABA***	F3-F4	1 µM
C2BA-4*	B5-B6	30 nM	Zearalanone***	F5-F6	1 µM
Pretilachlor***	B7-B8	100 nM	Benzyl butyl phthalate***	F7-F8	1 µM
ICI 172,780**	B9-B10	100 nM	TCPOBOP***	F9-F10	1 µM
Hyperforin***	B11-B12	100 nM	Cypermethrine***	F11-F12	3 µM
SR12813**	C1-C2	100 nM	2,4'DDT***	G1-G2	3 µM
Rifampicin**	C3-C4	300 nM	4,4'DDT***	G3-G4	3 µM
Oxadiazon***	C5-C6	300 nM	Fipronil sulfone***	G5-G6	3 µM
α-zearalanol***	C7-C8	1 µM	Ethinylestradiol**	G7-G8	3 µM
DEHP***	C9-C10	300 nM	Nonylphenol mixture***	G9-G10	3 µM
Clotrimazol***	C11-C12	300 nM	Bisphenol-B***	G11-G12	3 µM
Trans-nonachlor***	D3-D4	1 µM	Methoxychlor***	H3-H4	3 µM
RU43944****	D5-D6	1 µM	Bisphenol-A***	H5-H6	10 µM
4-OH-tamoxifen****	D7-D8	1 µM	Ferutinine**	H7-H8	10 µM
α-zearalenol***	D9-D10	300 nM	4-tert-cumylphenol***	H9-H10	10 µM

[#]For each ligand, the concentration used was determined prior to the screening experiment. It represents the dose at which the compound alone induces ≈30% of the luciferase expression obtained with 3 µM SR12813 (100% activation). *Compounds synthetized in the laboratory[4]. **Compounds purchased from Tocris Biosciences. ***Compounds purchased by Sigma Aldrich.****Gift from Sanofi Aventis. All compound stock solutions were prepared at 10 mM in DMSO.

Supplementary Table 2 | Clinical characteristics of the liver donors

Liver id	Sex	Age	Pathology
HH399	F	68	Metastasis of colorectal cancer
HH404	M	63	Organ donor
HH408	M	55	Organ donor

Supplementary Table 3 | Primer sequences

**CYP3A4**	Forward	TATTCTGTCTTCACAAACCG
	Reverse	TTTCTCACCAACACATCTCC
**GAPDH**	Forward	AATTGAGCCCGCAGCCTCCC
	Reverse	CCAGGCGCCCAATACGACCA

Supplementary References

1. Xue, Y. *et al.* Crystal structure of the pregnane X receptor-estradiol complex provides insights into endobiotic recognition. *Mol Endocrinol* **21**, 1028-1038 (2007).

2. Wang, W. *et al.* Construction and characterization of a fully active PXR/SRC-1 tethered protein with increased stability. *Protein Eng Des Sel* **21**, 425-433 (2008).

3. Chrencik, J.E. *et al.* Structural disorder in the complex of human pregnane X receptor and the macrolide antibiotic rifampicin. *Mol Endocrinol* **19**, 1125-1134 (2005).

4. Benod, C. *et al.* N-1H-benzimidazol-5-ylbenzenesulfonamide derivatives as potent hPXR agonists. *Bioorg Med Chem* **16**, 3537-3549 (2008).
